# Sexual Size Dimorphism in Rays and Skates (Elasmobranchii: Batoidea)

**DOI:** 10.1002/ece3.71858

**Published:** 2025-07-21

**Authors:** Joel H. Gayford, Scott G. Seamone, Duncan J. Irschick, Andrew Chin, Jodie L. Rummer

**Affiliations:** ^1^ College of Science and Engineering, James Cook University Townsville Queensland Australia; ^2^ Shark Measurements London UK; ^3^ Department of Marine Sciences Bahamas Agriculture and Marine Science Institute San Andros Bahamas; ^4^ Department of Biology Morrill Science Center, University of Massachusetts Amherst Massachusetts USA

**Keywords:** allometry, batoid, fecundity selection, Rensch's rule, reproductive mode, scaling, sexual selection, shark

## Abstract

Sexual size dimorphism (SSD) is a widely observed but poorly understood phenomenon in which male and female animals differ in body size (e.g., length or mass). Despite extensive research on the interspecific distribution of SSD across various lineages, the evolutionary drivers behind male‐biased and female‐biased SSD remain contentious. In Elasmobranchii (sharks and rays), it is hypothesised that spatiotemporal differences in reproductive effort distribution between oviparous and matrotrophic species underlie variation in the direction and magnitude of SSD. However, existing studies have focused almost exclusively on sharks, overlooking batoids (rays), which comprise over 50% of elasmobranch diversity. In this study, we analysed published size (total length and disc width) records from 187 batoid species to quantify interspecific SSD variation across batoids and tested for ecological and evolutionary drivers of SSD within a comparative phylogenetic framework. Our findings reveal that, although interspecific trends in SSD among batoids superficially mirror those in sharks, subtle differences emerge in ecological signal and modes of trait evolution between the two. These differences suggest that selection for substantial male‐biased and female‐biased SSD in batoids is weaker than in sharks. The underlying reasons for this remain unclear but may involve variation in fecundity selection between batoids and sharks. Further studies quantifying variation in sexual selection and fecundity selection will be essential to fully clarify the adaptive basis of SSD variation within elasmobranchs.

## Graphical abstract

Interspecific trends in sexual size dimorphism are superficially similar in batoids to sharks, but are underlain by different modes of evolution. Selection for substantial SSD in batoids seems to be weaker than in sharks. This may be due to differences in the strength of fecundity selection.

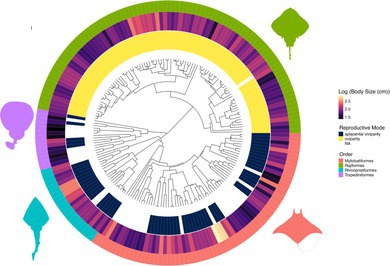

## 1 Introduction

### 1.1 Body Size and SSD

Body size (e.g., length or mass) is fundamentally important to animal ecology, behaviour and evolution (Schmidt‐Nielsen 1984). For this reason, research on correlates of body size, both in living animals and through deep time, has been a major focus of organismal research (Blanckenhorn 2000; Haldane 1926; Heim et al. 2015). One commonly studied aspect of body size is sexual size dimorphism (SSD), or body size differences between the sexes. SSD varies in both magnitude and direction (female‐biased vs. male‐biased) in different clades (Rohner et al. 2018; Webb and Freckleton 2007); however, our understanding of the adaptive drivers underpinning the evolution of SSD remains incomplete (Janicke and Fromonteil 2021; Tombak et al. 2024): Broadly speaking, it is thought that SSD results from a combination of sexual selection, fecundity selection and ecological selection during a species' evolution (Reeve and Fairbairn 1999; Serrano‐Meneses and Székely 2006; Shine 1989). The intensity of male–male competition (sexual selection) is often thought to modulate selection for large male size; whereas, female‐biased SSD may arise due to strong fecundity selection where larger females can carry more offspring (Butler et al. 2000; Cox et al. 2007; Reeve and Fairbairn 1999; Serrano‐Meneses and Székely 2006). However, observed trends may be complicated by the confounding effects of ecology—which can correlate with body size, sexual selection and fecundity selection (Maan and Seehausen 2011; Peters 1986; Pincheira‐Donoso and Hunt 2017; Shine 1989).

### 1.2 Methodological Issues Affecting Our Understanding of SSD

Our understanding of SSD is further complicated by methodological issues such as uneven taxon sampling and the use of coarse measures of dimorphism, which limit the robustness of some studies. For example, it has long been thought that in mammals, males are typically larger than females (Lindenfors et al. 2007). However, evolutionary studies of SSD in mammals have often relied on arbitrary distinctions between monomorphic and dimorphic taxa, while under‐representing the most speciose clades (Tombak et al. 2024). Revised estimates accounting for these limitations show that male‐biased SSD is not the norm in most mammal species (Tombak et al. 2024). Issues of taxonomic coverage, inconsistent measures of SSD and gaps in taxonomic coverage are widespread and extend beyond mammals. To fully understand the evolutionary basis of intraspecific body size variation and its links to ecology and behaviour, further studies investigating SSD across diverse taxonomic levels and lineages are essential (Blanckenhorn 2005; Shine 1989).

### 1.3 SSD in Elasmobranchii

Elasmobranchii (i.e., sharks, rays and skates) is an ideal clade in which to test hypotheses regarding the adaptive basis of SSD, as it exhibits both male‐ and female‐biased SSD (Gayford and Sternes 2024a) alongside significant variation in life history traits, particularly in reproductive biology and ecology (Conrath and Musick 2012; Ebert et al. 2021). Furthermore, sexual segregation and other sex‐based dietary preferences are widespread within Elasmobranchii (Ebert et al. 2021; Wearmouth and Sims 2010). Recent studies on cartilaginous fishes have revealed that oviparous species tend to exhibit weaker and less female‐biased SSD compared to viviparous species (Gayford and Sternes 2024a, 2024b). Notably, while male‐biased SSD is common across vertebrate taxa, female‐biased SSD predominates among sharks (Gayford and Sternes 2024a). However, Batoidea (i.e., rays and skates), which accounts for over 50% of elasmobranch diversity, has been excluded from these analyses. Despite their close phylogenetic relationship to sharks, batoids exhibit fundamental differences from most sharks, such as extreme dorsoventral flattening and the fusion of the pectoral fins to the head and anterior body (Martinez et al. 2016). Their ecological traits also differ significantly, with most batoids occupying benthic (i.e., associated with the substrate) habitats (Last et al. 2016). While there is no a priori reason to suggest that the unique morphology of batoids directly influences the interspecific distribution of SSD, body form and ecological lifestyle are known to constrain body size through established relationships with morphology, physiology and biomechanics (Ahti et al. 2020; Gidmark et al. 2019; Goldbogen 2018). Moreover, body form and habitat preferences have been found to influence fecundity and sexual selection across various lineages, including fishes (Bonduriansky and Rowe 2005; Gomes‐Jr and Monteiro 2007; Maan and Seehausen 2010, 2011; Zúñiga‐Vega et al. 2007). For example, the dorsoventrally flattened morphology of batoids could impose unique constraints on reproductive output relative to sharks. Given that fecundity and sexual selection are critical drivers of SSD across vertebrates (Reeve and Fairbairn 1999; Serrano‐Meneses and Székely 2006; Shine 1989), it is plausible that the nature of selection acting upon body size (and SSD) differs between batoids and sharks.

### 1.4 Study Aims

The purpose of this study was to examine systematic trends in SSD between batoids and other elasmobranchs, using published size records for 187 extant batoid species (Last et al. 2016). Here, we provide the first quantitative assessment of SSD across batoid diversity, testing for the presence of systematic trends in SSD distribution similar to those previously reported in sharks and other vertebrates. Specifically, we consider the potential influence of body size, reproductive mode, water depth and habitat preferences on SSD. Not only do these represent major axes of ecological variation in batoids (Last et al. 2016), but they provide a mechanism for testing relationships between SSD and ecology seen in other groups. For example, reproductive mode correlates with SSD in sharks, where oviparous species generally exhibit less pronounced (and more male‐biased) SSD compared to viviparous species (Gayford and Sternes 2024a). Additionally, water depth and habitat preferences may influence sexual selection and mate choice in fishes, as demonstrated in previous studies (Gomes‐Jr and Monteiro 2007; Maan and Seehausen 2010, 2011; Zúñiga‐Vega et al. 2007). Finally, the relationship between SSD and body size provides a means to evaluate Rensch's rule, which hypothesises that SSD should scale hypoallometrically with body size in taxa dominated by female‐biased SSD (Webb and Freckleton 2007). In other words, in taxa with female‐biased SSD, the magnitude of SSD should decrease with increasing body size.

Additionally, we reconstruct the evolutionary history of transitions in SSD direction and intensity within batoid phylogeny to explore whether early elasmobranchs exhibited minimal SSD relative to extant taxa (Gayford and Sternes 2024a). Given the broad morphological and ecological differences between batoids and sharks, we further hypothesise that relationships between ecological variation and SSD in batoids may differ from those reported in sharks. By comparing the results of this investigation with trends reported in sharks and other vertebrate clades, we provide novel insights into the evolutionary and ecological mechanisms underlying interspecific trends in SSD across vertebrate diversity.

## 2 Methodology

### 2.1 Data Collection

To quantify SSD in Batoidea, we extracted total length (i.e., disc length plus tail length) and disc width at sexual maturity for males and females of 187 extant species from the comprehensive reference guide Rays of the World (Last et al. 2016). The following procedure was initially applied to all batoid species found in Rays of the World. After excluding species for which size at sexual maturity for both sexes is unknown (rendering calculation of SSD metrics impossible; see data analyses section for further details), 187 species remained in our data set, covering all major radiations of extant batoids. While the Rays of the World dataset has certain limitations, including missing values and the lack of detailed information on intraspecific size variation, it remains the most extensive and reliable resource currently available, derived from published data. Unlike other databases such as Fishbase, Rays of the World contains consistent, standardised and verified body size data that has been collated by the same authors from scientific literature, reducing the potential for spurious errors and inaccuracies in the dataset. Total lengths were used wherever possible; however, where total length data was not available, disc width was used instead. For certain batoid lineages, particularly Myliobatiformes, disc width is more commonly reported as the standard measure of body size, despite recent evidence suggesting a tight correlation between the two measurements in some myliobatiform rays (Gayford et al. 2024). We considered the combination of disc width and total length values valid as both parameters reflect the major axis of body size variation in the taxa for which they are commonly used. For this reason, previous studies of body size evolution in Elasmobranchii have also used this approach (Pimiento et al. 2019). Additionally, maximum body size (either total length or disc width, as appropriate) was obtained for each species from the reference guide. Reproductive mode (oviparity or aplacental viviparity), habitat (demersal, bathydemersal, benthopelagic, reef‐associated and pelagic), minimum water depth (m) and maximum water depth (m) data for each species were also collected from FishBase (Froese and Pauly 2010). These variables were selected because they represent major ecological axes of variation in Elasmobranchii and have been considered as potential covariates of SSD in previous studies (Ebert et al. 2021; Gayford and Sternes 2024a, 2024b). As these values (and those from Rays of the World) represent average values for each species, it was not possible to assess intraspecific variation in SSD within batoid species in this study. The full set of 10,000 fully resolved phylogenetic trees from Stein et al. (2018) was downloaded to facilitate implementation of comparative phylogenetic methods. For comparative purposes, SSD data for nonbatoid elasmobranchs (sharks) was extracted from Gayford and Sternes (2024a).

### 2.2 Data Analyses

All analyses were performed in the R statistical environment (R Core Team 2024). To characterise SSD variation among species, two quantitative measures of SSD were calculated for each species, following the approach of Gayford and Sternes (2024a). The male‐to‐female ratio (MFR) was calculated as the ratio between size (total length or disc width) at sexual maturity in males and females, respectively. MFR represents a measure of the direction of SSD, where values greater than one indicate male‐biased SSD, values lower than one indicate female‐biased SSD and values of exactly one indicate the absence of SSD. Sexual dimorphism percentage (SD%) was calculated as the difference in size (total length or disc width) at sexual maturity between the sexes, expressed as a percentage of maximum body size. Consequently, SD% represents the magnitude of SSD (Gayford and Sternes 2024a).

**FIGURE 1:**
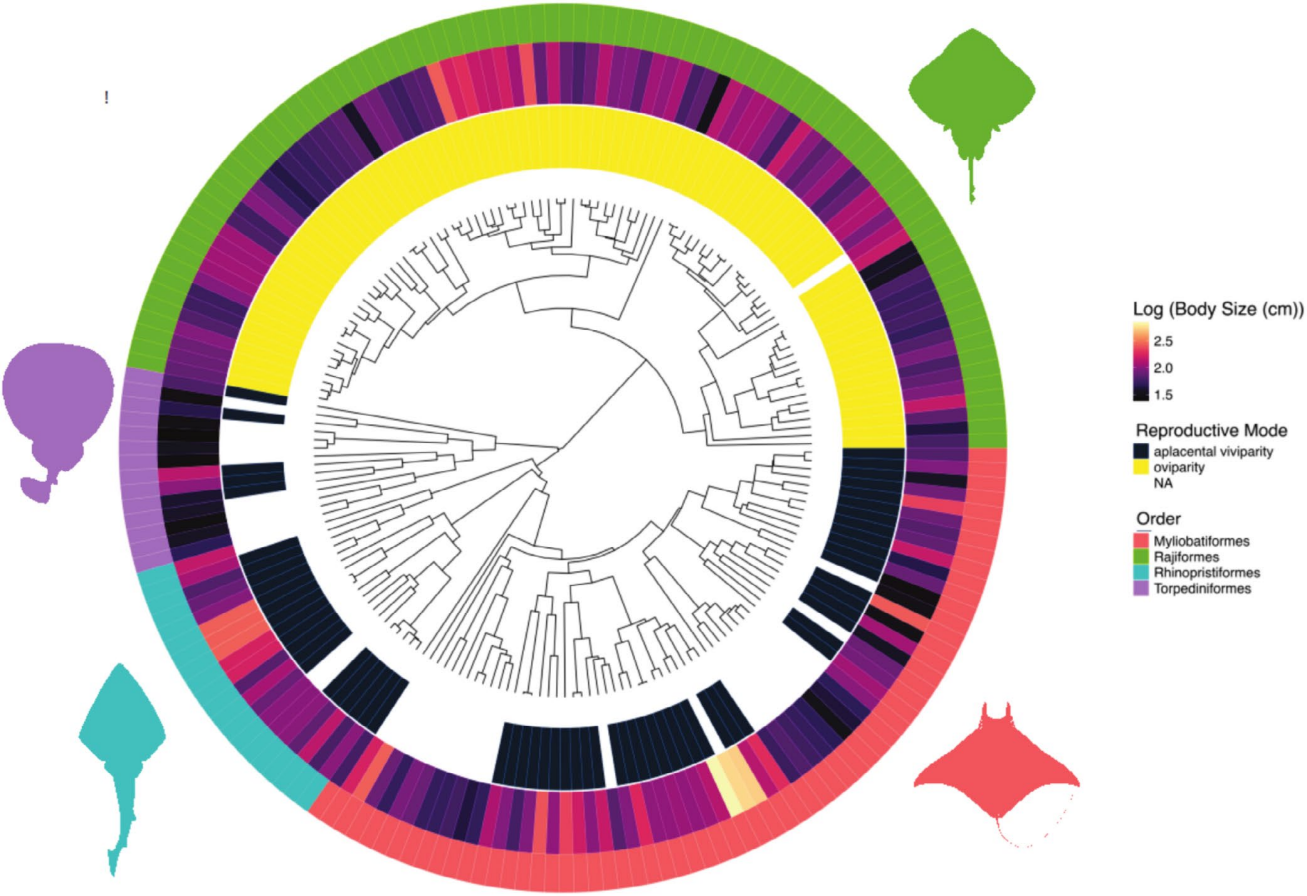
Maximum Clade Credibility (MCC) tree used for all analyses, highlighting variation in select traits (reproductive mode and maximum body size) among the batoid species in the final data set. ‘NA’ under reproductive mode indicates that data regarding the reproductive mode of a given taxon is absent from FishBase.

The phylogenetic interrelationships of various elasmobranch lineages are poorly resolved (Naylor et al. 2005; Villalobos‐Segura et al. 2022). To account for this phylogenetic uncertainty, a pruned maximum clade credibility (MCC) tree was inferred in the R package phangorn (Schliep 2011; Figure 1).

To explore the evolutionary dynamics shaping SSD distribution among batoid species, we applied a series of evolutionary models of continuous trait evolution to our SSD data set, using the maximum‐likelihood criterion in the R package mvMORPH (Clavel et al. 2015). For these, and all subsequent models, species with missing data (either SSD metrics or ecological values) were excluded from the relevant analyses. Specifically, four models were fit for each measure of SSD (MFR and SD%), each assuming one of the following covariance frameworks: Single‐peak Brownian Motion (BM1), multipeak Brownian Motion (BMM), single‐peak Ornstein–Uhlenbeck (OU1) and multipeak Ornstein–Uhlenbeck (OUM). Other phylogenetic covariance structures were not considered due to the lack of *apriori* information regarding the nature of body size evolution in batoids. Brownian motion represents a null model of trait evolution, a stochastic diffusion‐based process. Contrastingly, Ornstein–Uhlenbeck models represent directional evolution of traits towards one or more optima. Besides these modifications to the covariance framework, all other parameters were set to default, and models were compared based on corrected Akaike Information Criterion (AIC_c_) values.

To test for potential correlations between SSD and body size (Rensch's rule) and ecological variables, we fit a series of phylogenetic linear regression models in the R packages phylolm (Ho et al. 2016) and ape (Paradis and Schliep 2019). Six models were fit for each measure of SSD (MFR and SD%), with each model incorporating one of the following covariates: maximum body size, minimum water depth, maximum water depth or reproductive mode. Two additional models treated habitat as a covariate (details provided below). Each model incorporated the phylogenetic covariance matrix best supported by the results of the continuous trait evolution analyses (i.e., Brownian Motion or Ornstein–Uhlenbeck; see results for details). Maximum body size, minimum water depth and maximum water depth values were log‐transformed prior to model fitting. Reproductive mode was encoded as a binary variable, with oviparous species being assigned a value of zero and matrotrophic (i.e., aplacental viviparous) species assigned a value of one. Habitat data (ecotypes) were also encoded as a binary variable: demersal, bathydemersal and reef‐associated species assigned a value of zero, and pelagic species were assigned a value of one. Two separate habitat variables were defined for analysis: in the first (Habitat 1) benthopelagic species were assigned a value of one, while in the second (Habitat 2) benthopelagic species were assigned a value of zero.

To reconstruct the evolutionary history of SSD in Batoidea, ancestral state reconstruction of both MFR and SD% was implemented under a maximum‐likelihood criterion in the R package phytools (Revell 2024) using the MCC tree.

## 3 Results

**FIGURE 2:**
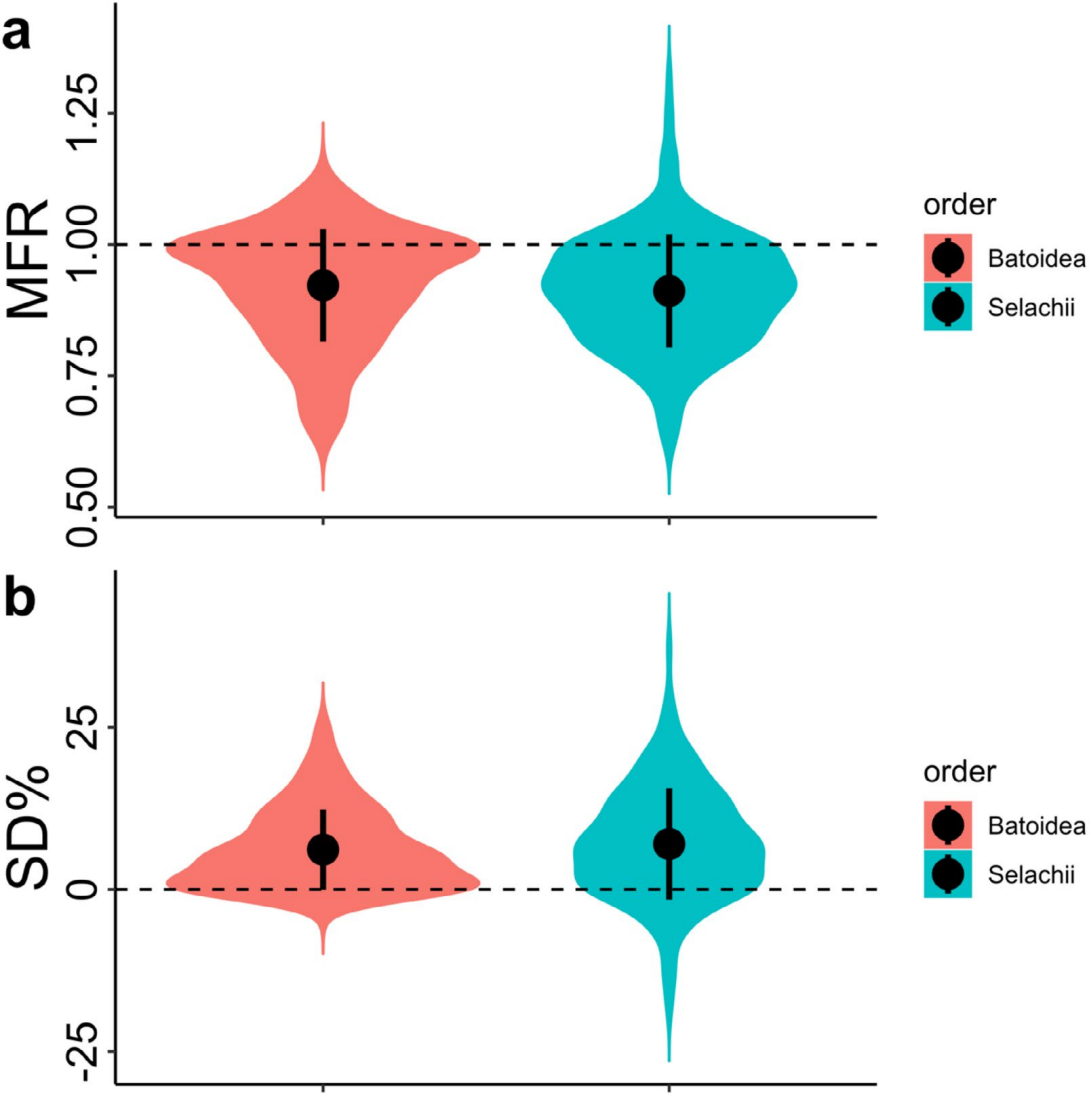
Distribution of male to female ratio (MFR) (a) and sexual dimorphism percentage (SD %) (b) values among batoid (Batoidea) and shark (Selachii) species. Black circles and bars represent overall mean values, and upper and lower quartiles.

There was no significant difference in mean MFR (male‐to‐female ratio) or SD% (sexual dimorphism percentage) values between sharks (MFR: 0.91 ± 0.11, SD%: 7.02 ± 8.58) and batoids (MFR: 0.92 ± 0.11, SD%: 6.15 ± 6.17), indicating that the most prevalent SSD system in both Batoidea and Selachii is slight, female‐biased SSD. While there was no difference between mean MFR or SD% values for Batoidea and Selachii, the distribution of values indicates that most batoids are less dimorphic in size than most sharks (Figure 2). Sharks also have a greater range of SSD values, achieving substantially higher (more male‐biased) MFR values and far more extreme SD% values than batoids (Figure 2).

Evolutionary model fitting indicated that the interspecific distribution of SD% values among batoids is best explained by a single‐peak Ornstein–Uhlenbeck model of trait evolution, with all other models performing significantly worse (Table 1). Contrastingly, the interspecific distribution of MFR values was best explained by a multipeak Brownian Motion model of trait evolution (Table 1).

**TABLE 1:** Model output for all models of continuous trait evolution, indicating likelihood and AIC values. ∆ AICc values indicate model support compared to the worst performing model, with the best performing model highlighted in bold.

SSD measure	Covariance model	Log likelihood	AIC_c_	∆ AIC_c_
SD%	BM1	−670.1	1344	—
SD%	BMM	−619.2	1247	97.69
SD%	OU1	**−600.7**	**1210**	**134.7**
SD%	OUM	−600.5	1213	131.0
MFR	BM1	475.1	−946.1	7.980
MFR	BMM	**479.3**	**−950.3**	**12.19**
MFR	OU1	473.2	−938.1	0.063
MFR	OUM	475.3	−938.1	—

Phylogenetic linear models revealed no significant correlations between SD% and water depth, reproductive mode, habitat or maximum body size (Table 2). In contrast, significant positive correlations were observed between MFR and maximum water depth, Habitat 1 and reproductive mode (Table 2). In the latter case, this indicates that oviparous taxa exhibit higher MFR values compared to aplacental viviparous taxa. Additionally, significant negative correlations were found between MFR and both maximum body size and Habitat 1 (Table 2). In the case of Habitat 1, this suggests that benthic taxa exhibit higher MFR values than pelagic or benthopelagic taxa.

**TABLE 2:** Output of phylogenetic linear regression models, including the estimated evolutionary parameters *α* and *σ* ^2^.

SSD measure	Covariate	Scaling coefficient	*t*	*p*	Log likelihood	AICc	*α*	*σ* ^2^
SD%	Minimum depth	0.082	0.290	0.773	−440.3	890.7	1.58e‐02	0.925
SD%	Maximum depth	−0.811	−1.691	0.093	−515.8	1042	1.16e‐02	0.543
SD%	Reproductive mode	−0.685	−0.220	0.826	−464.2	938.4	7.63e‐03	0.367
SD%	Habitat 1	0.010	0.008	0.994	−588.9	1187	0.010	0.439
SD%	Habitat 2	−0.912	−0.315	0.753	−588.8	1188	0.010	0.432
SD%	Body size	3.442	1.770	0.078	−587.3	1185	9.98e‐03	0.407
MFR	Minimum depth	0.005	0.968	0.335	134.1	−260.3	—	7.12e‐05
MFR	Maximum depth	0.022	3.385	0.001	138.6	−269.3	—	6.02e‐15
MFR	Reproductive mode	0.077	4.582	9.71e‐06	130.8	−253.6	—	4.11e‐15
MFR	Habitat 1	−0.491	−2.440	0.016	155.4	−302.8	—	6.96e‐16
MFR	Habitat 2	−0.022	−0.485	0.628	152.6	−297.1	—	5.42e‐16
MFR	Body size	−0.091	−2.964	0.003	156.8	−305.5	—	2.40e‐14

*Note:* *α* values are only given for models fit to SD% data, as these models assumed an Ornstein–Uhlenbeck covariance framework, unlike models fit to MFR data, which used a Brownian Motion covariance framework (Table 1).

**FIGURE 3:**
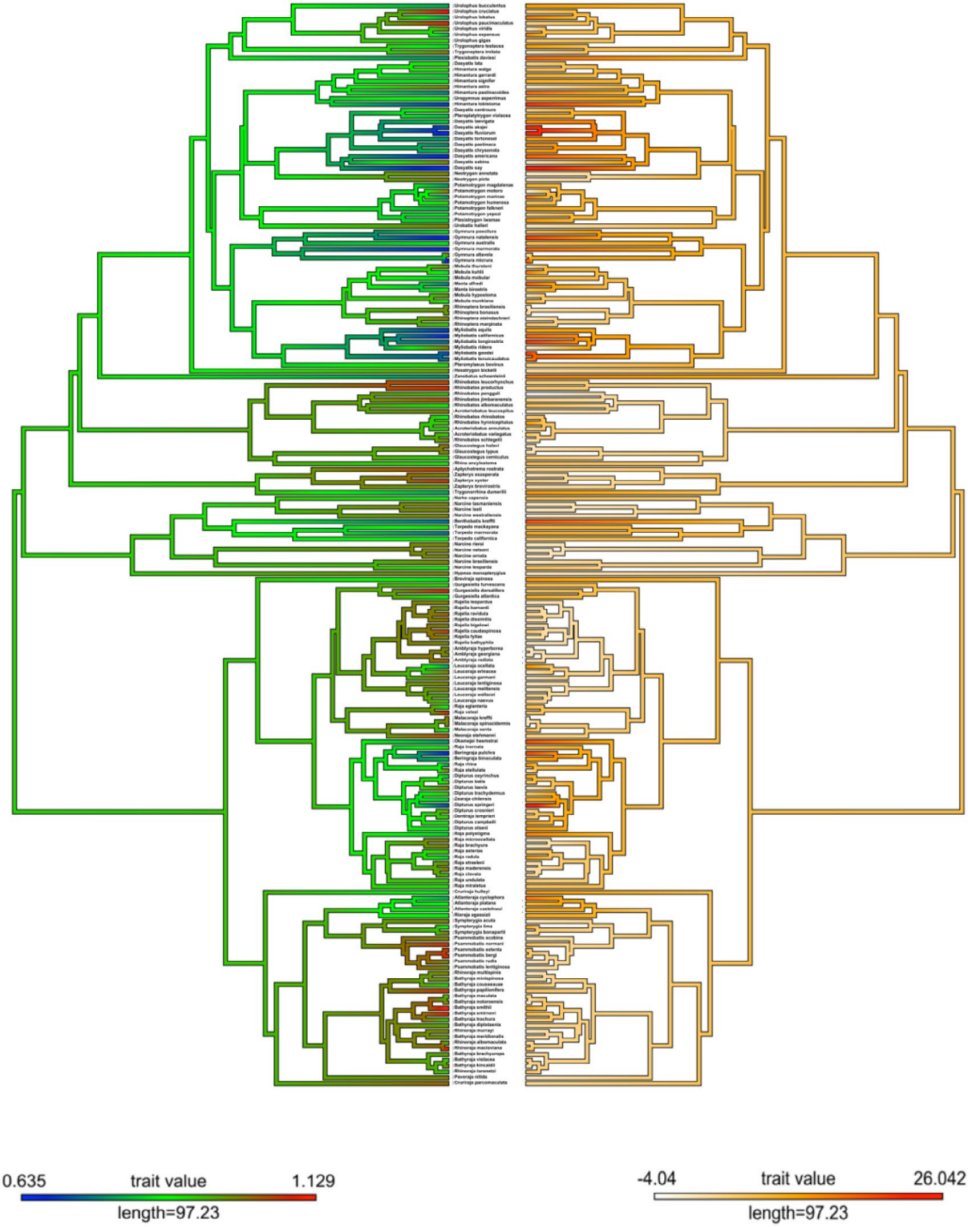
Relatively high and low MFR (left) and SD% (right) values have evolved independently within Batoidea. Tip labels match those provided in Stein et al. (2018).

Ancestral state reconstruction of both SD% and MFR indicated that evolutionary transitions towards relatively high and relatively low values have occurred on multiple independent occasions in both cases (Figure 3). We conservatively suggest that male‐biased SSD has evolved on a minimum of five occasions, and female‐biased SSD has evolved on a minimum of seven occasions (Figure 3). Moreover, our ancestral state reconstruction suggests that the most recent common ancestor of all batoids exhibited moderate female‐biased SSD, although it is not possible to rule out male‐biased SSD or even the absence of SSD in its entirety in early batoids (MFR: 0.92, 95% CI: 0.68 < x < 1.16; SD%: 6.21, 95% CI: 0 < x < 20.05).

## 4 Discussion

The purpose of this study was to quantify interspecific variation in SSD across living batoid diversity, identify potential ecological and life history correlates of SSD and compare these results to those found in sharks (and other vertebrates). The unique morphology of batoids and the interspecific distribution of ecological characteristics suggest that differences in the nature of selection underlying SSD between batoids and sharks are plausible. Moreover, the broader literature indicates that the drivers of interspecific variation in SSD may vary among different clades and across different taxonomic levels (Fairbairn et al. 2007; Janicke and Fromonteil 2021; Tombak et al. 2024). Overall, the distribution of SSD among batoids does not differ substantially from that observed in other vertebrates (Adkins‐Regan and Reeve 2014; Fairbairn et al. 2007; Janicke and Fromonteil 2021). As in some other vertebrate groups (e.g., Teleostei and Squamata), female‐biased SSD dominates (62% of species have female‐biased SSD), although male‐biased SSD has evolved independently on at least five occasions (Figure 3). However, as hypothesised, our results indicate that the evolutionary dynamics underlying interspecific variation in SSD among batoids and sharks may differ (Table 1; Table 2). Later in the discussion we consider in detail the ramifications of these differences, namely the ecological correlates of SSD and best supported models of trait evolution. Batoids and other elasmobranchs provide a unique opportunity to study the evolutionary dynamics of SSD in vertebrates given the sheer diversity of reproductive modes and ecological characteristics exhibited within the clade.

### 4.1 Macroevolutionary Trends in SSD

Clade‐wide assessments of SSD are critical to our understanding of macroevolutionary processes and morphological evolution through deep time (Cox et al. 2007; Jones and Sheard 2023). Indeed, our results indicate that there are several commonalities between sharks and batoids that have bearing on our broader understanding of macroevolutionary shifts in SSD among vertebrates. As in bony fishes, the majority of batoid and shark species show slight female‐biased SSD (Figure 2; Figure 3). Additionally, batoids clearly violate Rensch's rule, as the intensity of SSD does not scale hypoallometrically (i.e., the scaling coefficient of SD% with body size is not lower than 1) with body size (Table 2). Our ancestral state reconstruction also indicates that, as found in sharks (Gayford and Sternes 2024a), early batoids may not have exhibited SSD (Figure 3). This is not unexpected given that batoids are thought to be phylogenetically nested within sharks (Heinicke et al. 2009). However, the finding does give additional support to the notion of ancestral elasmobranchs either lacking SSD or exhibiting minor SSD relative to extant taxa. Given the key phylogenetic position of cartilaginous fishes as the sister group to all other jawed vertebrates (Criswell et al. 2017), any indication that early elasmobranchs lacked substantial SSD may prove valuable in future comparative studies addressing broad‐scale macroevolutionary shifts in SSD through vertebrate phylogeny. As originally hypothesised by Gayford and Sternes (2024a), an absence of significant SSD in early elasmobranchs may indicate that SSD observed in living species is a derived character state that has evolved independently of SSD in other vertebrate groups.

Importantly, our results do not provide conclusive evidence for the absence of SSD; however, they indicate that this ancestral state is equally as plausible as male‐biased or female‐biased SSD. The inclusion of fossil data can significantly alter the results of ancestral state reconstruction analyses (e.g., Finarelli and Flynn 2006), and hence it is possible that upon inclusion of fossil taxa exhibiting SSD, the support for SSD in ancestral elasmobranchs would increase substantially. This is not necessarily the case, however, as studies in other taxa (e.g., Jiménez‐Arcos et al. 2017) find support for ancestral states of substantial SSD, despite the omission of fossil data and substantial variation in SSD magnitude/direction among living species. Regrettably, the paucity of whole‐body fossils of batoid taxa makes the inclusion of extinct taxa in this study impossible. Even where rare whole‐body fossils do exist, they are not present in sufficient quantity to robustly determine size at maturity or maximum size ranges. While the results of this study provide insight into macroevolutionary trends in vertebrate SSD, the inclusion of novel phylogenetic and palaeontological data will be necessary to fully determine whether ancestral elasmobranchs were dimorphic in size or not.

### 4.2 Ecological Signal of SSD in Batoids

The influence of sexual selection, fecundity selection and other traits on SSD evolution is typically inferred through correlations between SSD magnitude/intensity and various ecological/life history variables (Fairbairn et al. 2007). While the magnitude of SSD in batoids is not affected by depth, habitat, reproductive mode or body size, there is a significant ecological signal underlying interspecific variation in SSD direction (Table 2). Specifically, smaller, oviparous (egg laying) and demersal species, and those present at greater depths are associated with greater MFR values (Table 2), and hence, are more likely to exhibit male‐biased SSD. Relationships between SSD, body size, habitat and altitude/water depth are commonplace across a number of vertebrate and invertebrate clades (Høye and Hammel 2010; Pincheira‐Donoso et al. 2021; Zhang et al. 2012); however, few studies have tested for these relationships in a marine context, making direct comparisons challenging. The trends linking SSD to body size and reproductive mode match those observed in sharks and likely result from relaxed selection for large female body size in oviparous species, as a result of their extended reproductive period and the external development of embryos (Gayford and Sternes 2024a, 2024b; Sims 2005). Indeed, the apparent relationship between reproductive mode and SSD direction may explain associations between MFR and depth/habitat too, given that oviparity is more prevalent in smaller, deeper species (Katona et al. 2023). The finding that only the direction (and not the magnitude) of SSD correlates with depth and habitat (Table 2) allows us to rule out resource partitioning as an alternative explanation, despite its role in shaping SSD trends in other taxa (Laliberté et al. 2022; Pearson et al. 2002). If resource partitioning between the sexes were the primary driver of SSD evolution in batoids, we might expect the magnitude of SSD to be greater in resource‐scarce environments (where resource partitioning may be more necessary to ensure persistence); however, this was not the case (Table 2).

However, ascribing all three systematic trends in batoid SSD direction purely to reproductive mode (or spatiotemporal variation in reproductive output) is unjustified without further research. Particularly given that the same logic should apply to sharks (Katona et al. 2023), which show no consistent relationship between depth and either direction or magnitude of SSD (Gayford and Sternes 2024a). Moreover, water depth and habitat preferences can profoundly influence sexual selection in bony fishes (Gomes‐Jr and Monteiro 2007; Maan and Seehausen 2010, 2011; Zúñiga‐Vega et al. 2007), a factor that can in turn help shape interspecific differences in SSD (Bernardy et al. 2024; Blanckenhorn 2005). While batoids may exhibit strong sperm competition and female mate choice (e.g., where females select preferred males for copulation, see Lyons et al. 2021), the sheer paucity of data regarding sexual selection in elasmobranchs makes it difficult to draw any conclusions as to the role of sexual selection in shaping interspecific trends in SSD among batoids (Rowley et al. 2019). In the case of habitat preferences, hydrodynamic and trophic differences between benthic and pelagic environments could influence selection for body size in batoids. The different dietary preferences of benthic or pelagic batoids may also affect the extent of sex‐based resource partitioning, a known driver of SSD (Laliberté et al. 2022; Pearson et al. 2002; Shine 1989). Even though our analyses indicate that resource partitioning is not the primary driver of SSD evolution in batoids, a more minor (yet still significant) role cannot be ruled out. Alternatively, where fecundity selection underlies interspecific SSD trends, the effects of elevated fecundity on body condition and fineness ratio may differ in their hydrodynamic significance between benthic and pelagic environments. However, there is no reason to suggest that these differences should consistently favour more female‐biased SSD in pelagic/benthopelagic batoids at present, particularly in the absence of any relationship between habitat and SSD magnitude (Table 2). Consequently, at present, spatiotemporal variations in reproductive output between oviparous and matrotrophic batoid species appear to be the primary factor determining interspecific differences in SSD, as in sharks.

Besides the three ecomorphological relationships described above, we also found that both measures of SSD scale either isometrically or hyperallometrically (with a scaling coefficient exceeding 1) with body size in batoids (Table 2), as observed in sharks (Gayford and Sternes 2024b). This result directly contradicts the prediction of Rensch's rule, a hypothesis that in taxa with female‐biased SSD, the magnitude of SSD should decrease with increasing body size (Webb and Freckleton 2007; Liao et al. 2015). Despite being referred to as a ‘rule’ it is becoming increasingly clear that Rensch's rule is the exception rather than the rule in taxa exhibiting female‐biased SSD (Halámková et al. 2013; Webb and Freckleton 2007), and hence our finding that SSD does not scale hypoallometrically in batoids (Table 2) is not unexpected. In line with previous studies in multiple vertebrate groups, it is likely that the violation of Rensch's rule in batoids results from relatively weak sexual selection for large males (Dale et al. 2007; Gayford and Sternes 2024b), although sexual selection for large females is also plausible. Additional empirical data on sexual selection in elasmobranchs will be required to further determine the validity of these hypotheses.

### 4.3 Fundamental Constraints on SSD That Are Absent in Sharks

Besides testing for ecological correlates and estimating past shifts in character state, insight into interspecific trends in SSD can be gained by comparing support for different models of trait evolution (Ceballos et al. 2012; Kuntner et al. 2019). Indeed, it is not just patterns of ecological signal in SSD that differ between sharks and batoids: Our results indicate that the evolutionary dynamics underlying the phylogenetic distribution of SD% and MFR values fundamentally differ between the two groups (Table 1). In sharks, the evolution of both the direction and magnitude of SSD is best explained by an Ornstein–Uhlenbeck model (Gayford and Sternes 2024a), whereas in batoids the direction of SSD is instead best explained by a Brownian Motion model (Table 1). While caution should be taken when interpreting Ornstein–Uhlenbeck models of trait evolution (Cooper et al. 2016), they are typically used to represent evolution towards one or more trait optima, consistent with the action of natural selection (Revell and Harmon 2022). Contrastingly, Brownian Motion is a diffusion‐based model of trait evolution that includes a substantial random component (Revell and Harmon 2022). Brownian Motion is not to be conflated with genetic drift, as despite this random component there are ways in which traits subject to strong selection can evolve under Brownian Motion (Hansen and Martins 1996). However, it is clear from our results that high MFR values observed in sharks are not observed in any of the batoid species included in this study (Figure 2). Moreover, MFR values are more uniformly centred around 1 (i.e., the absence of SSD) in batoids than in sharks (Figure 2). This, combined with evidence of Brownian Motion evolution, strongly implies that the nature (and possibly strength) of selection underpinning the direction of SSD differs between batoids and sharks. It should be noted that a plethora of phylogenetic covariance structures have been described (Cornwallis and Griffin 2024), and hence the models tested in this study do not necessarily represent the global models of best fit. However, in the absence of any prior information regarding how SSD evolves in batoids, these relatively simple models (Brownian Motion and Ornstein–Uhlenbeck) provide sufficient resolution to discern major differences between the adaptive landscapes of trait evolution in different clades. Further analyses would be necessary to provide finer‐scale information regarding differences in the nature of selection among elasmobranch subclades. This does not detract, however, from the key finding that SSD in sharks and rays, respectively, is best explained by different models of trait evolution (Table 1).

There is also evidence that the nature of selection acting on the magnitude of SSD differs between sharks and batoids (Table 1). While both are best explained by an Ornstein–Uhlenbeck model (consistent with directional evolution towards one or more trait optima, or ‘selective peaks’), a multioptimum model is preferred in sharks (Gayford and Sternes 2024a), as opposed to a single‐optimum model in batoids (Table 1). As with MFR values, SD% values in batoids are more uniformly centred around zero than in sharks (Figure 2), and both extremes of the distribution seen in sharks are absent in batoids (Figure 2). Therefore, we suggest that across batoid diversity, the predominant optimal phenotype is an absence of SSD, or negligible female‐biased SSD. This implies that selection for significant male or female‐biased SSD may be weaker in batoids than in sharks, potentially explaining the lack of significant correlations between ecological variables and the magnitude of SSD (Table 2). It is plausible that this results from the higher prevalence of oviparous taxa in this dataset and is thus a statistical artefact. However, it is also possible that fecundity selection for large female body size is relaxed in (oviparous) batoids relative to sharks. Polyembryony (also referred to as multiple embryos per eggcase, or MEPE), in which multiple embryos develop within a single egg capsule, has been documented in several batoid species (Chiquillo et al. 2014; Gayford 2025), but has yet to be documented as a viable reproductive strategy in sharks. The prevalence of this strategy remains unknown, but it does provide a potential mechanism of increasing fecundity without occupying additional space within the body cavity of the mother (Gayford 2025). Consequently, in taxa capable of polyembryony/MEPE, fecundity selection need not result in substantial female‐biased SSD. Other plausible mechanisms for altering the dynamics of fecundity selection between batoids and sharks could include differences in the temporal distribution of reproductive effort (frequency of reproductive cycles), or differences in the hydrodynamic consequences of increased fecundity between different elasmobranch body forms. However, the necessary data to discriminate between these hypotheses is absent from the literature.

## 5 Conclusions and Future Directions

Characterising the ecological correlates and evolutionary dynamics underlying SSD in a range of lineages improves our ability to understand the extent to which superficially similar phenotypic patterns (e.g., female‐biased or male‐biased SSD) can arise under distinct selective regimes. In the case of elasmobranchs, batoids exhibit superficially similar interspecific trends in SSD to sharks (Figures 2 and 3; Gayford and Sternes 2024a), although SSD is generally more pronounced in the latter (Figure 2). The direction of SSD in batoids, as in sharks, appears to be shaped by spatiotemporal differences in the distribution of reproductive effort between matrotrophic and oviparous species (Table 2). However, there are also unexplained correlations between the direction of SSD and both water depth and habitat preferences that warrant further study. In particular, if these relationships reflect true signals of ecological selection (as opposed to artefacts of potential relationships between ecology and reproductive mode), our results may provide insight into selection of body size and population dynamics across spatial and environmental gradients.

Additionally, the evolutionary dynamics underlying both the magnitude and direction of SSD appear to differ between batoids and sharks. When and why these shifts in evolutionary dynamics occurred remains unknown, and further studies are required to better quantify interspecific differences in fecundity selection and sexual selection among elasmobranchs. In particular, accurate body size information is absent in the literature for hundreds of extant batoid species and should form a major focus of future work. Further compounding this data deficiency is a lack of comprehensive data regarding the body size of extinct batoid fishes. The uncertainty associated with ancestral state reconstruction analyses is elevated substantially where fossil data (the only direct evidence of past phenotypic change) cannot be incorporated. If we wish to fully understand past macroevolutionary shifts in SSD both within Elasmobranchii and between elasmobranchs and other vertebrates, this limitation must be overcome through comprehensive descriptions of ontogeny and maximum body sizes in extinct batoids. Furthermore, while some studies linking maternal size to reproductive effort do exist (e.g., Kume et al. 2009), robust information regarding how reproductive output scales with size within and across batoid species is needed to refine our understanding of female‐biased SSD in particular.

An additional question that remains unanswered is the potential consequences of SSD for conservation and management. Batoids face significant anthropogenic threats including overfishing and habitat destruction (Dulvy et al. 2021). If population declines resulting from these threats are size‐specific, SSD could be crucial in determining long‐term population stability. However, literature linking SSD to population viability and management is scarce, even in other taxa, necessitating further work in this area.

Overall, the results presented in this study indicate that superficially similar patterns of SSD among shark and ray species may be underlain by discrete evolutionary trends. This adds to the broader SSD literature indicating that interspecific patterns of sex‐based size differences result from a complex interplay of ecological, sexual and fecundity selection (Fairbairn et al. 2007). Despite these similarities, our evolutionary analyses also indicate that the SSD observed in extant elasmobranchs may have arisen independently of that observed in other major vertebrate lineages. Consequently, the results presented here will provide a valuable datapoint for future comparative studies investigating broad‐scale evolutionary trends in SSD through vertebrate phylogeny.

## Author Contributions

**Joel H. Gayford:** conceptualization (lead), data curation (lead), formal analysis (lead), methodology (lead), writing – original draft (lead), writing – review and editing (lead). **Scott G. Seamone:** conceptualization (supporting), writing – original draft (supporting), writing – review and editing (supporting). **Duncan J. Irschick:** writing – original draft (supporting), writing – review and editing (supporting). **Andrew Chin:** writing – review and editing (supporting). **Jodie L. Rummer:** writing – original draft (supporting), writing – review and editing (supporting).

## Conflicts of Interest

The authors declare no conflicts of interest.

## Data Availability

All data and code necessary to replicate the results of this study have been placed in the following repository: https://doi.org/10.6084/m9.figshare.28579565.
